# Parallel Loss of Plastid Introns and Their Maturase in the Genus *Cuscuta*

**DOI:** 10.1371/journal.pone.0005982

**Published:** 2009-06-19

**Authors:** Joel R. McNeal, Jennifer V. Kuehl, Jeffrey L. Boore, Jim Leebens-Mack, Claude W. dePamphilis

**Affiliations:** 1 Department of Plant Biology, University of Georgia, Athens, Georgia, United States of America; 2 Department of Biology, Huck Institutes of the Life Sciences, and Institute of Molecular Evolutionary Genetics, The Pennsylvania State University, University Park, Pennsylvania, United States of America; 3 DOE Joint Genome Institute and Lawrence Berkeley National Laboratory, Walnut Creek, California, United States of America; 4 Genome Project Solutions, Hercules, California, United States of America; CNRS UMR 8079 / Université; Paris-Sud, France

## Abstract

Plastid genome content and arrangement are highly conserved across most land plants and their closest relatives, streptophyte algae, with nearly all plastid introns having invaded the genome in their common ancestor at least 450 million years ago. One such intron, within the transfer RNA *trnK*-UUU, contains a large open reading frame that encodes a presumed intron maturase, *matK*. This gene is missing from the plastid genomes of two species in the parasitic plant genus *Cuscuta* but is found in all other published land plant and streptophyte algal plastid genomes, including that of the nonphotosynthetic angiosperm *Epifagus virginiana* and two other species of *Cuscuta*. By examining *matK* and plastid intron distribution in *Cuscuta*, we add support to the hypothesis that its normal role is in splicing seven of the eight group IIA introns in the genome. We also analyze *matK* nucleotide sequences from *Cuscuta* species and relatives that retain *matK* to test whether changes in selective pressure in the maturase are associated with intron deletion. Stepwise loss of most group IIA introns from the plastid genome results in substantial change in selective pressure within the hypothetical RNA-binding domain of *matK* in both *Cuscuta* and *Epifagus*, either through evolution from a generalist to a specialist intron splicer or due to loss of a particular intron responsible for most of the constraint on the binding region. The possibility of intron-specific specialization in the X-domain is implicated by evidence of positive selection on the lineage leading to *C. nitida* in association with the loss of six of seven introns putatively spliced by *matK*. Moreover, transfer RNA gene deletion facilitated by parasitism combined with an unusually high rate of intron loss from remaining functional plastid genes created a unique circumstance on the lineage leading to *Cuscuta* subgenus *Grammica* that allowed elimination of *matK* in the most species-rich lineage of *Cuscuta*.

## Introduction

Introns within organellar genes have unique features. Unlike those in eukaryotic nuclear genes, they do not rely on spliceosomes for excision from RNA transcripts, and unlike the similarly structured self-splicing introns of prokaryotes, they typically require other trans-acting factors for efficient splicing *in vivo* [1], [2]. Land plant plastid genomes usually contain between 17 and 20 of these introns, all of which are classified as group II based on putative folding structure except for a single group I intron within the transfer RNA gene *trnL*-UAA[3]. Plastid group II introns are further subdivided structurally into two classes, group IIA and group IIB. All of these group II introns, with the exception of the second of two group II introns within *clpP*, seemingly trace their origin to a shared common ancestor of charophycean algae and all land plants[4].

Only one transcribed open reading frame has been identified within any plastid intron, the presumed intron maturase *matK*, consistently found within *trnK*-UUU. Although *matK* has been shown to be an essential factor for the splicing of the *trnK* intron within which it is contained[5], its involvement in the splicing of other plastid introns is poorly understood[6]. The plastid genome of the nonphotosynthetic, parasitic angiosperm *Epifagus* encodes only four proteins not involved in transcription or translation and lacks a functional *trnK* gene[7]. However, the *trnK* pseudogene retains a complete open reading frame for *matK* which is evolving under selective constraint, indicating *matK* is essential for other functions beyond splicing the *trnK* intron in that species[8]. A parallel pattern of *trnK* loss with retention of *matK* is seen in the photosynthetic streptophyte alga *Zygnema circumcarinatum* [4]. Various studies have shown that without translation of plastid-encoded proteins, seven group IIA introns in the plastid genome remain in an unspliced transcript form, whereas group IIB introns are largely unaffected and have been shown in maize to primarily rely upon a nuclear-encoded factor, *crs2*, for splicing[3], [9], [10]. An eighth group IIA intron, *clpP* intron 2, is present in the chloroplast genomes of most land plants but was not examined in those studies because it is not present in grasses. Excision of the only group I intron, *trnL*-UAA, is unaffected by any of these factors, as is splicing of the second of two group IIB introns found within *ycf3* [10]. Reliance of seven group IIA introns upon a plastid-encoded factor for splicing indicates a role for *matK* in splicing introns other than the *trnK* intron within which it resides.

Like *Epifagus* (Orobanchaceae), members of the genus *Cuscuta* (Convolvulaceae) are parasitic plants that have undergone substantial gene loss from their plastid genomes [11]. However, at least some members of the genus retain a largely intact plastid genome and contain chlorophyllous tissues [12], albeit in a localized form less crucial to the parasites' survival relative to fully autotrophic plants[13]. Losses of three group IIA introns from the plastid genomes of various *Cuscuta* species were reported more than a decade ago [14], [15], and presence of the intron found within the 3′ locus of the *trans*-spliced *rps12* gene was shown to be polymorphic in the genus[16]. More recently, the sequencing of four complete plastid genomes from the genus *Cuscuta*, two from subgenus *Monogyna* and two from subgenus *Grammica*, shows that intron content between the two subgenera differs greatly; specifically, both *matK* and all group IIA introns except the second intron of *clpP* are lost from the plastid genomes of the closely related members of subgenus *Grammica* [11], [17]. Intron 2 of *clpP*, acquired in the common ancestor of land plants millions of years after *matK* and the other seven plastid group IIA introns [4], was shown to be properly transcribed and translated in *Cuscuta gronovii* in the absence of plastid *matK* [17].

In this study we sampled across the taxonomic range of *Cuscuta* in order to ascertain the distribution of *matK* and plastid introns in the genus. For those taxa that still contain *matK*, we investigated whether or not significant changes in selective constraint occurred on branches where intron loss has occurred. Finally, we conducted similar branchXsite tests on an equal sample size of the variously parasitic family Orobanchaceae, where loss of most plastid introns is known to have occurred at least in *Epifagus*.

## Results

Using PCR assays that gave clear positive or negative results based on band size, we surveyed for the presence of *matK* at the *trnK*-UUU locus along with all known group IIA introns and three group IIB introns (one in *trnG*-UCC and two within *ycf3*) from a variety of *Cuscuta* species representing all three currently recognized subgenera (Table 1). In cases of tRNA introns, we used sequence reads to confirm presence or absence of the gene and intron, as tRNA exons are generally shorter than 40 nucleotides in length.

**Table 1:** Intron distribution in relevant taxa.

Taxon	Subgenus	Group IIA								Group IIB	
		*trnK*-UUU	*atpF*	* *trnV*-UAC	*rpl2*	3′*rps12*	*trnI*-GAU	*trnA*-UGC	*clpP* intron 2	*trnG*-UCC	*ycf3* (both)
*Nicotiana tabacum*		+	+	+	+	+	+	+	+	+	+
*Ipomoea purpurea*		+	+	+	−	+	+	+	+	+	+
*Cuscuta exaltata*	*Monogyna*	×	+	+/×	−	+	+	+	+	+	+
*C. reflexa*	*Monogyna*	×	+	+/×	−	+	+	+	+	+	+
*C. japonica*	*Monogyna*	×	+	+/×	−	+	+	+	+	+	+
*C. lupuliformis*	*Monogyna*	×	+	+/×	−	+	+	+	+	+	+
*C. europaea*	*Cuscuta*	×	+	×	−	+	+	+	+	×	+
*C. epilinum*	*Cuscuta*	×	+	×	−	+	+	+	×	×	+
*C. nitida*	*Cuscuta*	×	−	×	−	+	×	×	+	×	−
*C. indecora*	*Grammica*	×	−	×	−	−	×	×	+	×	−
*C. umbellata*	*Grammica*	×	−	×	−	−	×	×	+	×	−
*C. tasmanica*	*Grammica*	×	−	×	−	−	×	×	+	×	−
*C. rostrata*	*Grammica*	×	−	×	−	−	×	×	+	×	−
*C. gronovii*	*Grammica*	×	−	×	−	−	×	×	+	×	−
*C. obtusiflora*	*Grammica*	×	−	×	−	−	×	×	+	×	−
*Epifagus*		×	×	×	+	+	×	×	+	×	×

* trnV introns in *Cuscuta* subg. *Monogyna* have deletions that may render them pseudogenes.

Intron presence or absence is shown for *Nicotiana tabacum* (Solanaceae), *Ipomoea purpurea* and *Cuscuta* spp. (Convolvulaceae), and *Epifagus virginiana* (Orobanchaceae). *Nicotiana*, *Ipomoea*, and *Cuscuta* are classified in the order Solanales, while *Epifagus virginiana* is in the closely related order Lamiales. Subgeneric taxonomic classifications are listed for *Cuscuta* spp. *Nicotiana* intron distribution is typical of most angiosperms. “+” indicates intron present, “−” indicates precise intron loss from an intact gene, and “X” indicates loss of gene (and intron) from the plastid genome. Intron data for *Nicotiana*, *Ipomoea*, *Cuscuta exaltata*, *Cuscuta reflexa*, *Cuscuta gronovii*, *Cuscuta obtusiflora*, and *Epifagus* were gleaned from complete genome sequences available on genbank; all other data are based on PCR and PCR sequencing assays.

Although the *trnK* gene itself is absent across all *Cuscuta* species, all sampled members of subgenus *Monogyna* and subgenus *Cuscuta* retain an open reading frame for *matK*, paralleling the condition in *Epifagus* and *Zygnema*. However, all sampled members of subgenus *Grammica*, which contains the majority of *Cuscuta* species, have lost *matK* from the plastid genome. As predicted under the hypothesis that *matK* is necessary for splicing of all seven group IIA introns shown to be unspliced in grass plastid translational mutants [3], [9], [10], loss of *matK* in *Cuscuta* correlates perfectly with the loss of all of those group IIA introns from the plastid genome. Representatives of subgenus *Grammica* still possess the group IIA intron within *clpP* (intron 2), five group IIB introns, and the *trnL*-UAA group I intron within otherwise normal genes, corroborating prior results that resident plastid *matK* is not necessary for the splicing of these introns [3], [5], [9], [10], [17].

**Figure 1:**
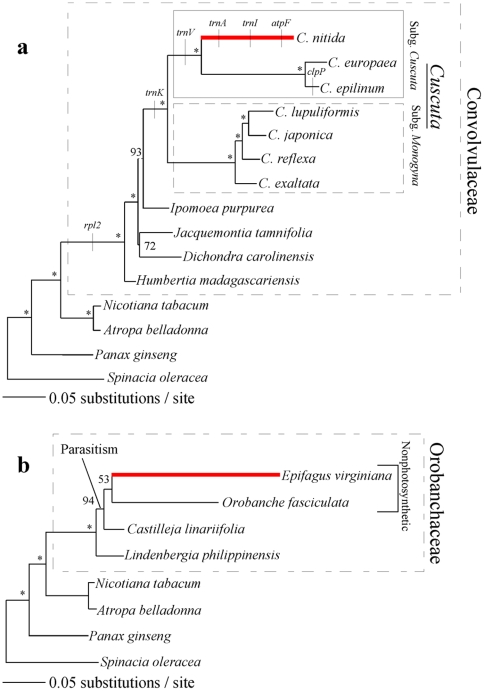
Phylogenies of Convolvulaceae (a) and Orobanchaceae (b) inferred from full and partial nucleotide sequences of *matK*. Branches shown in red denote significant increases in selection within domain X of *matK*. Trees were produced using Maximum Likelihood with bootstrap values (100 replications) shown at the nodes (bootstrap values of 100 are denoted by asterisks). Taxonomic delimitations of *Cuscuta* subgenera and Convolvulaceae are boxed and labeled. Group IIA intron losses are mapped on branches where they are inferred to have occurred. The branch joining *Ipomoea* with *Cuscuta* species, denoted with a dotted line, was collapsed for analysis due to low support in other studies.

All sampled species of *Cuscuta* that still possess *matK* also possess at least four group IIA introns with the exception of *Cuscuta nitida*, which retains only the 3′ *rps12* intron (Table 1, Fig. 1A) and intron 2 of *clpP*. The open reading frame of *matK* was partially or fully sequenced for five species in *Cuscuta* subgenus *Monogyna*, three species from subgenus *Cuscuta*, and four species from the otherwise autotrophic family they are derived from within, Convolvulaceae (Morning Glory Family). Using outgroup sequences from available plastid genomes, a well-supported phylogeny was constructed that agrees fully with published relationships within Convolvulaceae and *Cuscuta* [18], [19] (Fig. 1A). *Ipomoea* (tribe Convolvuleae) was strongly supported as sister to *Cuscuta*, although alternative hypotheses at this node could not be rejected in a previous study study[20]. Because our taxon sampling outside of *Cuscuta* is sparse, we conservatively chose to collapse this node as a polytomy for analyses of selective constraint. We were especially interested in changes in selective constraint within domain X, the portion of *matK* that has been identified as the putative RNA binding domain [21]. When per-site ratios of nonsynonymous to synonymous nucleotide substitutions (*d_N_*/*d_S_* = *ω*) were constrained across the phylogeny, domain X was found to be evolving under stronger purifying selection (*ω* = 0.21) than the remainder of the gene (*ω* = 0.392; Table 2, model M0). All sampled species also contained an amino acid consensus motif within domain X (SX_3–6_TLAXKXK) conserved across land plants and charophytes[22], further suggesting that *matK* remains functional among all *Cuscuta* that still possess it.

**Table 2:** Shifting patterns of selection on *matK*.

Model (parameters)	Omega ω_0_, ω_1_, ω_2_	-ln (Likelihood)	Model Comparison*	LRT statistic (df, p)
**A. ** ***Cuscuta nitida*** ** lineage - X domain**				
1. M0 (29)	0.21	1440.306	---	---
*2.* **M3 (31)**	**0.089, 0.681**	**1423.843**	**M0 vs M3**	**32.926 (2, <<0.001)**
**3. Discrete BranchXSites (33)**	**0.067, 0.623, 4.582**	**1418.591**	**M3 vs discrete bXs**	**10.505 (2, 0.005)**
4. M1a, Nearly Neutral (30)	0.115, 1.0	1425.3	---	---
5. M2a, Pos. Sel. (32)	0.115, 1.0, 1.0	1425.3	M1a vs M2a	0 (2,1)
6. Pos. Sel BranchXSites null§ (31)	0.094, 1.0, 1.0	1421.596	---	---
7. **Pos. Sel BranchXSites (32)**	**0.094, 1.0, 4.371**	**1420.719**	**M1a vs Pos. Sel. bXs**	**9.161 (2, 0.01)**
8.			Null§ vs Pos. Sel. bXs	1.753 (0∶1, >0.5)
**B. ** ***Cuscuta nitida*** ** lineage nonX-domain regions**				
9. M0 (29)	0.392	4875.979	---	---
10. M3 (31)	0.202, 0.854	4839.185	M0 vs M3	73.588 (2,<<0.001)
11. Discrete BranchXSites (33)	0.207, 0.875, 0	4838.633	M3 vs Discrete bXs	1.103 (2, 0.576)
12. M1a, Nearly Neutral (30)	0.233, 1.0	4839.67	---	---
13. M2a, Pos. Sel. (32)	0.238, 1.0, 2.66	4839.206	M1a vs M2a	0.927 (2, 0.629)
14. Pos. Sel BranchXSites null§ (32)	0.233, 1.0, 1.0	4839.67	---	---
15. Pos. Sel BranchXSites (32)	0.233, 1.0, 1.0	4839.67	M1a vs Pos. Sel. bXs	0 (2, 1)
16.			Null§ vs Pos. Sel. bXs	0 (0∶1, 1)
**C. ** ***Epifagus virginiana*** ** lineage X-domain**				
17. M0 (16)	0.2823	990.106	---	---
18. **M3 (18)**	**0.055, 0.535**	**985.246**	**M0 vs M3**	**9.719 (2, 0.008)**
19. **Discrete BranchXSites (20)**	**0, 0.454, 4.077**	**980.044**	**M3 vs Discrete bXs**	**10.405 (2, 0.006)**
20. M1a, Nearly Neutral (17)	0.204, 1.0	985.919	---	---
21. M2a, Pos. Sel. (19)	0.17218, 1.0, 1.0	985.919	M1a vs M2a	0
22. Pos. Sel BranchXSites null§ (18)	0.1, 1.0, 1.0	983.387	---	---
23. **Pos. Sel BranchXSites (19)**	**0.115, 1.0, 4.54**	**982.839**	**M1a vs Pos. Sel. bXs**	**6.144 (2, 0.046)**
24.			Null§ vs Pos. Sel. bXs	1.097 (0∶1, >0.05)
**D. ** ***Epifagus*** ** nonX-domain**				
25. M0 (16)	0.47	4228.341	---	---
26. **M3 (18)**	**0.315, 1.43**	**4203.435**	**M0 vs M3**	**49.812 (2, <<0.001)**
27. **Discrete BranchXSites (20)**	**0.311, 1.385, 14.885**	**4194.264**	**M3 vs Discrete bXs**	**18.342 (2, <0.001)**
28. M1a, Nearly Neutral (17)	0.255, 1.0	4204.865	---	---
29. M2a, Pos. Sel. (19)	0.27, 1.0, 4.773	4202.478	M1a vs M2a	4.774 (2, 0.092)
30. Pos. Sel BranchXSites null§ (18)	0.237, 1.0, 1.0	4203.087	---	---
31. **Pos. Sel BranchXSites (19)**	**0.261, 1.0, 14.427**	**4195.222**	**M1a vs Pos. Sel. bXs**	**19.286 (2, <<0.001)**
32.			**Null** § **vs Pos. Sel. bXs**	**15.73 (0∶1, <<0.001)**

Likelihood Ratio Tests indicate shifts in pattern of selection on *Cuscuta nitida* (Convolvulaceae) and *Epifagus virginiana* (Orobanchaceae) *matK* genes following loss of all but one (3′*rps12* in *Cuscuta nitida*) or two (3′*rps12* and *rpl2* in *Epifagus virginiana*) group IIA introns from the plastid genome. Models and parameters are described in text. Models with significantly improved likelihoods relative to null hypothesis are shown in boldface. Clade and branch descriptions refer to relationships depicted in Figure 1.

* Models M0, M1a, M2a, and M3 are described in text with branchXsites (bXs) models for M3 and M1a.

§ Null model 2 for positive selection is BranchXSites model constraining foreground ω_2_ to neutrality (ω_2_ = 1.0).

Significant variation in selective constraint across sites within domain X was observed when comparing nested models with a single ratio of *d_N_*/*d_S_* (M0) versus models with two or three rate ratio classes (M3; Table 2, line 2). We used *fitmodel* [23] to test whether changes in the pattern of among-site variation in selective constraint varied across the tree, perhaps in association with the loss of specific group II introns (Fig. 1). A Likelihood Ratio Test (LRT) did not yield significantly better support for a model that allowed switching among rate ratio classes across the tree relative to the M3 model (among-site variation in *d_N_*/*d_S_* ) without switching across the tree (*p* = 0.32; Fig. 1). We wanted to explore this further by focusing on the branch leading to *C. nitida*, which has lost all introns that are thought to be spliced by *matK* (see above) with the exception of the one contained in the 3′ portion of *rps12*. We used branchXsites models implemented in *codeml* [24], [25], [26] to test the *a priori* hypothesis that the pattern of variation in constraint among sites was different on the branch leading to *C. nitida* (specified as the foreground branch) than the pattern of among-site variation across the rest of the tree (background branches). This approach is analogous to the switching test implemented in *fitmodel*, but in this case we have an *a priori* hypothesis that switches among rate ratio classes are concentrated on a single branch. The alternative hypothesis for branchXsites tests of Yang, Nielson and colleagues (implemented in *codeml*) have fewer parameters than the unconstrained switching model implemented in *fitmodel*, and thus this approach may have more statistical power when one has well defined hypothesis for the branch on which switching is expected to have occurred. In fact, the branchXsites model fits the domain X data significantly better than the rates across sites model (M3) when the *C. nitida* branch was specified as the foreground (Tables 2, line 3, and 3, test 1). By contrast, the likelihood for discrete branchXsite model was not significantly different than the rates across sites model (M3) when residues outside of domain X were analyzed (Table 2, line 11).

**Table 3:** Results of Branch-sites analyses.

Site Class*	0	1	2a	2b	Pos. Selec. Sites** (posterior prob.)
**1. ** ***Cuscuta nitida*** ** M3 bXs (X domain)**					
Proportion of sites	0.56562	0.22014	0.15421	0.06002	
Background ω	0.06745	0.62299	0.06745	0.62299	
*C. nitida* lineage ω	0.06745	0.62299	4.58222	4.58222	
**2. ** ***Cuscuta nitida*** ** Positive Selection bXs (X domain)**					
Proportion of sites	0.62212	0.17038	0.16288	0.04461	16 E (0.981)
Background ω	0.0938	1	0.0938	1	
*C. nitida* lineage ω	0.0938	1	4.37149	4.37149	
**3. ** ***Epifagus virginiana*** ** M3 bXs (X domain)**					
Proportion of site	0.33139	0.37839	0.1355	0.15472	
Background ω	0	0.45392	0	0.45392	
*E. virginiana* lineage ω	0	0.45392	4.07717	4.07717	
**4. ** ***Epifagus virginiana*** ** Positive Selection bXs (X domain)**					
Proportion of site	0.58225	0.16591	0.19599	0.05585	None with pp>0.95
Background ω	0.11534	1	0.11534	1	
*E. virginiana* lineage ω	0.11534	1	4.5397	4.5397	
**5. ** ***Epifagus virginiana*** ** M3 bXs (nonX-domain regions)**					
Proportion of site	0.75647	0.18138	0.05013	0.01202	
Background ω	0.31107	1.38574	0.31107	1.38574	
*E. virginiana* lineage ω	0.31107	1.38574	14.88588	14.88588	
**6. ** ***Epifagus virginiana*** ** Positive Selection bXs (nonX-domain regions)**					
Proportion	0.65688	0.27813	0.04566	0.01933	130 P (0.968), 177 F (0.951)
Background ω	0.26096	1	0.26096	1	
*E. virginiana* lineage ω	0.26096	1	14.42669	14.42669	

Foreground (*Cuscuta nitida* or *Epifagus virginiana* lineages) and background omega (ω = *dN/dS*) parameters for Branch-sites models with significant LRT results (Table 2).

* Background and foreground values of ω_0_, ω_1_ and ω_2_ listed in the table.

** Sites implicated as evolving under positive selection in Positive Selection bXs model (Bayes empirical Bayes posterior probabilities >0.95 [25] in tests of positive selection on foreground branches (Table 2) listed as position in alignment and derived amino acid residue.

Returning to analyses of domain X, two branchXsites tests were designed specifically to detect evidence of adaptive evolution[25], [26]. The first test compares the “nearly neutral” model (M1a) with codons evolving under conserved (0<ω_0_<1) and neutral (ω_1_ = 1) evolution, with a positive selection branchXsites model that includes a third, positive rate ratio class (ω_2_>1) for a fraction of sites evolving on the foreground branch. The second, more stringent test compares a branchXsites null model with ω_2_ = 1 on the foreground branch to the positive selection branchXsites model (i.e. ω_2_>1 on the foreground branch). In addition, *codeml* [27] provides *a posteriori* Bayes empirical Bayes (BEB) estimation of the probability that each site on the foreground branch is evolving under positive selection (ω_2_>1). The likelihood for the branchXsites positive selection model was significantly better than for the nearly neutral model, and adaptive evolution on the branch leading to *C. nitida* was strongly supported for one site, position 16 in the domain X alignment (Tables 2, line 7, and 3, test 2). However, we were unable to reject a more stringent null model (ω_2_ = 1; Table 2, line 8). In summary, these results indicate that loss of three of the final four group IIA introns for which *matK* has been implicated in splicing has resulted in relaxed or even positive selection for some codons within domain X in *C. nitida*.

In *Epifagus* one of only two remaining, putatively *matK*-spliced plastid group IIA introns is the same 3′ *rps12* intron retained in *C. nitida*; the second is an intron in *rpl2* that is not found in any *Cuscuta* species nor autotrophic relatives in Convolvulaceae[28]. Because *Epifagus* retains only one additional intron relative to *Cuscuta nitida*, we used *codeml* to perform LRT analyses testing whether *matK* may also be evolving under positive selection in Orobanchaceae, the predominantly parasitic family containing *Epifagus*. Although knowledge of intron distribution among members of Orobanchaceae is lacking, we gathered *matK* data from a range of species available on Genbank that likely differ in plastid gene and intron content from *Epifagus*. *Orobanche fasciculata*, like *Epifagus*, is nonphotosynthetic but is known to retain a possibly functional copy of *rbcL*, the large subunit of the Rubisco protein crucial to the Calvin Cycle[29]. A parasite that retains the ability to photosynthesize (*Castilleja linariifolia*) and a fully autotrophic sister-group to the parasites (*Lindenbergia philippinensis*) were also included in the analysis, and the same outgroups were used as for the Convolvulaceae tests. The phylogeny obtained for these species (Fig. 1B) was congruent with published relationships; although the branch joining the two nonphotosynthetic Orobanchaceae sensu strictu taxa, *Orobanche fasciculata* and *Epifagus*, has relatively low support in our tree, this relationship is incontrovertibly supported in all other systematic work done on Orobanchaceae to date [30], [31], [32]. As was the case with the *Cuscuta*/Convolvulaceae result, global *d_N_*/*d_S_* (ω in M0) was lower for domain X than for the rest of the gene (0.28 vs. 0.47), and branchXsites models with the *Epifagus* lineage set as the foreground were significantly better than the rates across sites models (M1a and M3; Table 2, line 18). As was also seen in the *Cuscuta*/Convolvulaceae analysis, positive selection was implicated when the nearly neutral model was set as the null, but not when the more stringent null model (ω_2_ = 1; Tables 2, lines 23 and 24, and 3, test 4) was imposed. Unlike the *Cuscuta*/Convolvulaceae analysis, however, both *fitmodel* and *codeml* analyses identified shifting levels of constraint across branches for some sites outside of domain X and strong evidence for positive selection on the branch leading to *Epifagus* (Tables 2, lines 31 and 32).

## Discussion

In the evolutionary history of *Cuscuta*, the previously conserved RNA-binding domain of *matK* underwent dramatic change in selective pressure after the loss of three of the remaining four group IIA introns for which *matK* is involved in splicing. In *Cuscuta nitida*, the RNA-binding domain is evolving under less constraint than in other *Cuscuta* species and outgroups where multiple group IIA introns spliced by *matK* are still present. It is possible that constraint on domain X to remain a generalist for group IIA intron binding has been released on the branch leading to *Cuscuta nitida*, and *matK* may have subsequently specialized to specifically bind to and splice the 3′ *rps12* intron. Alternatively, one of the three introns lost on the branch to *Cuscuta nitida* may be particularly integral to maintaining constraint on domain X. Results of the branchXsites analyses are suggestive of adaptive evolution in domain X on the *Cuscuta nitida* lineage, but not conclusive. While the Maximum Likelihood estimations of ω_2_ were >4.0 for some codons on the *Cuscuta nitida* lineage, we are not able to reject the hypothesis that these sites are evolving under neutrality (ω_2_ = 1.0; Table 2, lines 7 and 8). This may be due to insufficient statistical power.

*Epifagus*, which retains two group IIA introns linked to *matK* splicing in its plastid genome, also shows a dramatic change in selective constraint of domain X relative to related taxa. If one of the three introns lost on the branch leading to *Cuscuta nitida* (*trnF*-GAU, *trnA*-UGC, and *atpF*) is primarily responsible for constraint of domain X across streptophytes, that intron may be lost on the branch leading to *Epifagus* as well. As we saw with the *Cuscuta* analysis, the Maximum Likelihood estimations of ω were >4.0 for some codons in domain X; however, we are not able to reject the hypothesis that these sites are evolving under neutrality (ω_2_ = 1.0; Table 2, lines 23 and 24). Interestingly, we were able to reject neutral evolution for some sites in the amino terminal region, outside of domain X on the branch leading to *Epifagus* (Table 2, line 32). The sites showing significant signal for positive selection (Table 3, test 6) are moderately conserved in the pfam alignment for the matK amino terminal region (positions 224 and 277 in the complete alignment of pfam01824), but no function has been hypothesized for this portion of the matK protein.

Loss of tRNA genes is a common phenomenon in the plastid genomes of parasitic plants[33], [34], [35], and *Epifagus* has also lost the group IIA-containing *atpF* gene along with all other photosynthetic and chlororespiratory genes[7]. However, there are no cases of intron loss from functional genes in *Epifagus*. Although sampled members of subgenus *Grammica* parallel *Epifagus* in losing all group IIA intron-containing tRNAs, *atpF* and *rps12* remain under purifying selection in *Cuscuta* despite precise intron losses from these genes. Intron 2 of *clpP*, a group IIA intron not linked to *matK* splicing, was uniquely lost by *Cuscuta epilinum* (Table 1); that species still retains *clpP* intron 1, a group IIB intron. However, the group IIB introns in *ycf3* are also precisely lost from subgenus *Grammica* and *Cuscuta nitida* (Table 1), indicating a mechanism for intron loss that is not limited to group IIA introns. Intron losses from intact plastid genes are not unprecedented in land plants[14], [36], [37], but they are sporadic and rare. Such losses are much more frequent in conjugating charophycean algae, perhaps due to higher rates of homologous recombination or levels of reverse transcriptase activity[4]. Independent loss of six introns from five different functional genes in *Cuscuta* suggests this lineage is much more prone to purge introns from its plastid genomes than other land plants, although the mechanism for this increased rate of intron loss is unclear. Because the *rpl2* intron was lost before the evolution of parasitism in *Cuscuta* [28], the high rate of intron loss from otherwise intact genes in *Cuscuta* may or may not be related to its parasitic habit.

Loss of *matK* from the plastid genome of *Cuscuta* is only possible due to a unique combination of tRNA loss related to heterotrophy and a predisposition for plastid intron loss that is otherwise unknown in land plants. This special situation provides an opportunity to test the prediction that *matK* is indeed required for splicing of most group IIA introns, but isn't required for the evolutionarily distinct group IIA intron 2 of *clpP*, group IIB introns, nor the group I intron in *trnL*-UAA. Since the invasion of the chloroplast genome by all group II introns other than intron 2 of *clpP* at least 450 million years ago, *matK* has performed the role of both a *cis-* and *trans*- group IIA intron-splicing element in the plastid genome. All plastid genomes retaining any of these group IIA introns in genes necessary for survival must also retain a functional copy of *matK*; thus, loss of *matK* from functional plastid genomes is expected to be rare or perhaps even nonexistent in land plants other than *Cuscuta*. Parallel changes in *matK* associated with intron loss in two independent lineages of parasitic plants indicate that reduction of generalist splicing requirements may cause the protein to undergo adaptive changes to specialize on remaining intron splicing functions. Alternatively, one of three introns lost on the branch to *Cuscuta nitida* and possibly also on the branch to *Epifagus* may be primarily responsible for the high constraint of the RNA-binding domain of *matK*. Investigation of these and other parasitic lineages, which have evolved as natural plastid gene and intron knockout mutants, will help further understanding of organellar intron and maturase coevolution.

## Materials and Methods

Complete plastid genome sequences of *Cuscuta obtusiflora*, *Cuscuta exaltata*, and *Ipomoea purpurea* were used to design primers for this study, assess presence of non-group IIA introns within *Cuscuta*, to eliminate the possibility of gene transpositions in cases of PCR-detected intron and *matK* loss, and to verify the presence of only the expected loci for genes examined in this study. Genbank accession numbers and voucher numbers for sequences used for this study are shown in Table 4.

**Table 4:** Voucher information and *matK* GenBank accession numbers.

Species	Voucher #	Genbank accession
*Cuscuta exaltata*	*	NC009963
*C. reflexa*	#	EU330285
*C. japonica*	#	EU330283
*C. lupuliformis*	(PAC) JRM03.0808	EU330284
*C. europaea*	(PAC) JRM03.1101	EU330282
*C. epilimum*	(PAC) JRM03.1210a	EU330281
*C. nitida*	*	EU330280
*C. indecora*	(PAC) JRM03.1103	*matK* absent
*C. umbellata*	*	*matK* absent
*C. tasmanica*	*	*matK* absent
*C. rostrata*	(PAC) JRM03.1001	*matK* absent
*C. obtusiflora*	(PAC) JRM03.0207	*matK* absent
*Ipomoea purpurea*	(PAC) JRM03.1203	NC009808
*Jacquemontia tamnifolia*	(MO) 00883399	EU330286
*Dichondra carolinensis*	#	EU330287
*Humbertia madagascariensis*	(MO) 3854462	EU330288
*Nicotiana tabacum*	N/A	**NC001879**
*Atropa belladona*	N/A	**NC004561**
*Epifagus virginiana*	N/A	**NC001568**
*Orobanche fasciculata*	N/A	**AF051990**
*Castilleja linariifolia*	N/A	**AF051981**
*Lindenbergia philippinensis*	N/A	**AF051994**
*Panax ginseng*	N/A	**NC006290**
*Spinacia oleracea*	N/A	**NC002202**

Sequences generated outside of our group are shown in bold. Specimens that lacked enough material for herbarium voucher are denoted by an asterisk (*); photographs of the dissected flowers used for identification are available upon request. Plant material or DNA from other labs where no voucher information was provided were verified by sequence identity to existing vouchered sequences on GenBank and are marked with a #.

Primer combinations to assay intron or *matK* presence were chosen for ease of band size interpretation on 1% agarose gels stained with ethidium bromide. PCRs for *matK* and plastid introns were conducted using a combination of published [30], [38], [39], [40] and newly designed primer sequences (Table 5). Most sequencing was performed on a Beckman-Coulter CEQ8000 system according to manufacturers protocol, and the remaining sequences were generated by the Pennsylvania State University Nucleic Acids Facility on an ABI 3730XL.

**Table 5:** Primer sequences designed for this study.

atpF-F	5′-ATGAAMRACGTAACCKATT-3′
atpF-R	5′-CTCTTTGTAAGGYTTGTTG-3′
ycf3-F	5′-TCAGGAGAAAAAGAGGCATT-3′
ycf3-R	5′-GCAATTTCAGAATCTCCCTGTTG-3′
rrn16-endF	5′-GTGAAGTCGTAACAAGGTAGCCG-3′
rrn23-R1	5′-CGTCTCTGGGTGCCTAGGTATCC-3′
clpP-1F	5′-ATGCCYATTGGTGTTCCAARAG-3′
clpP-C562R	5′-CCCCTACAACATCRACAAKTCC-3′
trnKConv-endF	5′-CACTATGTATCATTTGATAACCC-3′
matKConv-54F	5′-CCTATATCCACTTMTCTTTCAGGAG-3′
matKConv-783F	5′-GTYTTTGYTAAGGATTTTMAGG-3′
matKConv-801F	5′-GGCCAACCTAGGCTTGCTCAAGG-3′
matKConv-882R	5′-TTGAAGCCAGAAKKGATTTTCC-3′
matKConv-1339R	5′-AGTTCKAGCRCAAGAAAG-3′
matKConv-1423R	5′-GTTCTTCCGACGTWAAGAATTCTTC-3′
matKConv-1450F	5′-TTTRTATCRAATAAAGTATATAC-3′
trnKsubgM-F1	5′-GGGCGAGTATAAAGAGAGAGGG-3′
matKsubgM-2R	5′CGTTCAATAATATCAGAATCT-3′
matKsubgM-3F	5′-CGCGCTTTTTTACAAAGCTTGGG-3′
matKsubgM-ex3R	5′-CCCAAGCTTTGTAAAAAAGCGCG-3′
matKsubgM-ex4F	5′-ATCTCAGAATTTACGATCAATTC-3′
matKsubgM-ex5R	5′-TGTAGAAAGAATTGTAATAAATG-3′
matKsubgM-ex6R	5′-CGAAGCGTCTTGTACCCAGACCG-3′
matKsubgC-R1	5′-GAATCTGAKAARTCGGYCCAACC-3′
matKsubgC-R2	5′-CAMGATTTCCARATGAGGGGGG-3′

*matK* primers designed using sequence from subgenus *Monogyna* are designated by the suffix subgM, ones designed using subgenus *Cuscuta* sequences by subgC, and ones designed with Convolvulaceae sequences by Conv.

Separate *matK* phylogenies were estimated for the *Cuscuta*/Convolvulaceae and *Epifagus*/Orobanchaceae analyses. Maximum Likelihood (ML) trees were estimated in PAUP*4.0b10 [41] using GTR + gamma models with parameters estimated from the data. The ML trees were used in molecular evolutionary analyses to test for change in constraint on lineages leading to *Cuscuta nitida* and *Epifagus*. Likelihood ratio tests were applied to compare a series of nested models including equal constraint (M0 *d_N_/d_S_* = ω), variation in ω across sites (M3, M2a and 1a ) and distinct patterns of variation across sites on foreground and background branches (branchXsites models). Model parameter and likelihood values (Table 2 and 3) were estimated using *codeml* within the PAML package v.3.15 [27]; http://abacus.gene.ucl.ac.uk/software/paml.html). Foreground branches were specified as those leading to *Cuscuta nitida* or *Epifagus* in separate analyses. Sites with Bayes empirical Bayes posterior probabilities >0.95 for ω_2_>1.0 were estimated in *codeml* [25].

We also checked for switching among ω rate ratio classes across the Convolvulaceae and Orobanchaceae trees using *fitmodel* v0.5.2 program [42] www.cebl.auckland.ac.nz/~sguindon/fitmodel.html]. Unlike codeml, *fitmodel* does not specify foreground and background branches.

## References

[1] Lambowitz AM, Perlman PS (1990). Involvement of aminoacyl-transfer RNA-synthetases and other proteins in group-I and group-Ii intron splicing.. Trends in Biochemical Sciences.

[2] Michel F, Umesono K, Ozeki H (1989). Comparative and functional anatomy of group II catalytic introns- a review.. Gene.

[3] Vogel J, Borner T, Hess WR (1999). Comparative analysis of splicing of the complete set of chloroplast group II introns in three higher plant mutants.. Nucleic Acids Research.

[4] Turmel M, Otis C, Lemieux C (2005). The complete chloroplast DNA sequences of the charophycean green algae *Staurastrum* and *Zygnema* reveal that the chloroplast genome underwent extensive changes during the evolution of the Zygnematales.. BMC Biology.

[5] Vogel J, Hubschmann T, Borner T, Hess WR (1997). Splicing and intron-internal RNA editing of *trnK*-*matK* transcripts in barley plastids: Support for *matK* as an essential splice factor.. Journal of Molecular Biology.

[6] Liere K, Link G (1995). RNA-binding activity of the *matK* protein encoded by the chloroplast *trnK* intron from Mustard (*Sinapis alba* L.).. Nucleic Acids Research.

[7] Wolfe KH, Morden CW, Palmer JD (1992). Function and evolution of a minimal plastid genome from a nonphotosynthetic parasitic plant.. Proceedings of the National Academy of Sciences of the United States of America.

[8] Young ND, dePamphilis CW (2000). Purifying selection detected in the plastid gene *matK* and flanking ribozyme regions within a group II intron of nonphotosynthetic plants.. Molecular Biology and Evolution.

[9] Hubschmann T, Hess WR, Borner T (1996). Impaired splicing of the *rps12* transcript in ribosome-deficient plastids.. Plant Molecular Biology.

[10] Jenkins BD, Kulhanek DJ, Barkan A (1997). Nuclear mutations that block group II RNA splicing in maize chloroplasts reveal several intron classes with distinct requirements for splicing factors.. Plant Cell.

[11] McNeal JR, Kuehl JV, Boore JL, dePamphilis CW (2007). Complete plastid genome sequences suggest strong selection for retention of photosynthetic genes in the parasitic plant genus *Cuscuta*.. BMC Plant Biology.

[12] Haberhausen G, Valentin K, Zetsche K (1992). Organization and sequence of photosynthetic genes from the plastid genome of the holoparasitic flowering plant *Cuscuta reflexa*.. Molecular & General Genetics.

[13] Hibberd JM, Bungard RA, Press MC, Jeschke WD, Scholes JD (1998). Localization of photosynthetic metabolism in the parasitic angiosperm *Cuscuta reflexa*.. Planta.

[14] Downie SR, Olmstead RG, Zurawski G, Soltis DE, Soltis PS (1991). Six independent losses of the chloroplast DNA *rpl2* intron in Dicotyledons - Molecular and phylogenetic implications.. Evolution.

[15] Bommer D, Haberhausen G, Zetsche K (1993). A large deletion in the plastid DNA of the holoparasitic flowering plant *Cuscuta reflexa* concerning two ribosomal-proteins (*rpl2*, *rpl23*), one transfer-RNA (*trnI*) and an *orf2280* homolog.. Current Genetics.

[16] Freyer R, Neckermann K, Maier RM, Kossel H (1995). Structural and functional-analysis of plastid genomes from parasitic plants - Loss of an intron within the genus *Cuscuta*.. Current Genetics.

[17] Funk HT, Berg S, Krupinska K, Maier UG, Krause K (2007). Complete DNA sequences of the plastid genomes of two parasitic flowering plant species, *Cuscuta reflexa* and *Cuscuta gronovii*.. BMC Plant Biology.

[18] Neyland R (2001). A phylogeny inferred from large ribosomal subunit (26S) rDNA sequences suggests that *Cuscuta* is a derived member of Convolvulaceae.. Brittonia.

[19] McNeal JR, Arumuganathan K, Kuehl JV, Boore JL, dePamphilis CW (2007). Systematics and plastid genome evolution of the cryptically photosynthetic parasitic plant genus *Cuscuta* (Convolvulaceae).. BMC Biology.

[20] Stefanovic S, Olmstead RG (2004). Testing the phylogenetic position of a parasitic plant (*Cuscuta*, Convolvulaceae, Asteridae): Bayesian inference and the parametric bootstrap on data drawn from three genomes.. Systematic Biology.

[21] Mohr G, Perlman PS, Lambowitz AM (1993). Evolutionary relationships among groupII intron-encoded proteins and identification of a conserved domain that may be related to maturase function.. Nucleic Acids Research.

[22] Sanders ER, Karol KG, McCourt RM (2003). Occurrence of *matK* in a *trnK* group II intron in charophyte green algae and phylogeny of the Characeae.. American Journal of Botany.

[23] Guindon S, Rodrigo AG, Dyer KA, Huelsenbeck JP (2004). Modeling the site-specific variation of selection patterns along lineages.. Proceedings of the National Academy of Sciences of the United States of America.

[24] Yang Z, Nielsen R (2002). Codon-substitution models for detecting molecular adaptation at individual sites along specific lineages.. Molecular Biology and Evolution.

[25] Yang Z, Wong WS, Nielsen R (2005). Bayes empirical bayes inference of amino acid sites under positive selection.. Molecular Biology and Evolution.

[26] Zhang J, Nielsen R, Yang Z (2005). Evaluation of an improved branch-site likelihood method for detecting positive selection at the molecular level.. Molecular Biology and Evolution.

[27] Yang Z (2007). PAML 4: phylogenetic analysis by maximum likelihood.. Molecular Biology and Evolution.

[28] Stefanovic S, Krueger L, Olmstead RG (2002). Monophyly of the Convolvulaceae and circumscription of their major lineages based on DNA sequences of multiple chloroplast loci.. American Journal of Botany.

[29] Wolfe AD, dePamphilis CW (1998). The effect of relaxed functional constraints on the photosynthetic gene *rbcL* in photosynthetic and nonphotosynthetic parasitic plants.. Molecular Biology and Evolution.

[30] Young ND, Steiner KE, dePamphilis CW (1999). The evolution of parasitism in Scrophulariaceae/Orobanchaceae: Plastid gene sequences refute an evolutionary transition series.. Annals of the Missouri Botanical Garden.

[31] Olmstead RG, dePamphilis CW, Wolfe AD, Young ND, Elisons WJ (2001). Disintegration of the Scrophulariaceae.. American Journal of Botany.

[32] Bennett JR, Mathews S (2006). Phylogeny of the parasitic plant family Orobanchaceae inferred from Phytochrome A.. American Journal of Botany.

[33] Taylor GW, Wolfe KH, Morden CW, Depamphilis CW, Palmer JD (1991). Lack of a functional plastid transfer RNA(Cys) gene is associated with loss of photosynthesis in a lineage of parasitic plants.. Current Genetics.

[34] Wimpee CF, Morgan R, Wrobel RL (1992). Loss of transfer-RNA genes from the plastid 16S-23S ribosomal-RNA gene spacer in a parasitic plant.. Current Genetics.

[35] Lohan AJ, Wolfe KH (1998). A subset of conserved tRNA genes in plastid DNA of nongreen plants.. Genetics.

[36] Downie SR, Llanas E, KatzDownie DS (1996). Multiple independent losses of the *rpoC1* intron in angiosperm chloroplast DNA's.. Systematic Botany.

[37] McPherson MA, Fay ME, Chase MW, Graham SW (2004). Parallel loss of a slowly evolving intron from two closely related families in asparagales.. Systematic Botany.

[38] Dumolin-Lapegue S, Pemonge MH, Petit RJ (1997). An enlarged set of consensus primers for the study of organelle DNA in plants.. Molecular Ecology.

[39] Demesure B, Sodzi N, Petit RJ (1995). A set of universal primers for amplification of polymorphic noncoding regions of mitochondrial and chloroplast DNA in plants.. Molecular Ecology.

[40] Nickrent DL, Yan OY, Duff RJ, dePamphilis CW (1997). Do nonasterid holoparasitic flowering plants have plastid genomes?. Plant Molecular Biology.

[41] Swofford DL (2002). PAUP*.Phylogenetic Analysis Using Parsimony (*and Other Methods).

[42] Guindon S, Rodrigo AG, Dyer KA, Huelsenbeck JP (2004). Modeling the site-specific variation of selection patterns along lineages.. Proceedings of the National Academy of Sciences of the United States of America.

